# **β**-Secretases, Alzheimer's Disease, and Down Syndrome

**DOI:** 10.1155/2012/362839

**Published:** 2012-02-28

**Authors:** Robin L. Webb, M. Paul Murphy

**Affiliations:** ^1^Department of Molecular and Cellular Biochemistry, University of Kentucky, Lexington, KY 40536-0230, USA; ^2^The Sanders-Brown Center on Aging, University of Kentucky, Lexington, KY 40536-0230, USA; ^3^The UK Center on Muscle Biology, University of Kentucky, Lexington, KY 40536-0230, USA

## Abstract

Individuals with Down Syndrome (DS), or trisomy 21, develop Alzheimer's disease (AD) pathology by approximately 40 years of age. Chromosome 21 harbors several genes implicated in AD, including the amyloid precursor protein and one homologue of the **β**-site APP cleaving enzyme, BACE2. Processing of the amyloid precursor protein by **β**-secretase (BACE) is the rate-limiting step in the production of the pathogenic A**β** peptide. Increased amounts of APP in the DS brain result in increased amounts of A**β** and extracellular plaque formation beginning early in life. BACE dysregulation potentially represents an overlapping biological mechanism with sporadic AD and a common therapeutic target. As the lifespan for those with DS continues to increase, age-related concerns such as obesity, depression, and AD are of growing concern. The ability to prevent or delay the progression of neurodegenerative diseases will promote healthy aging and improve quality of life for those with DS.

## 1. Introduction

**Figure 1:**
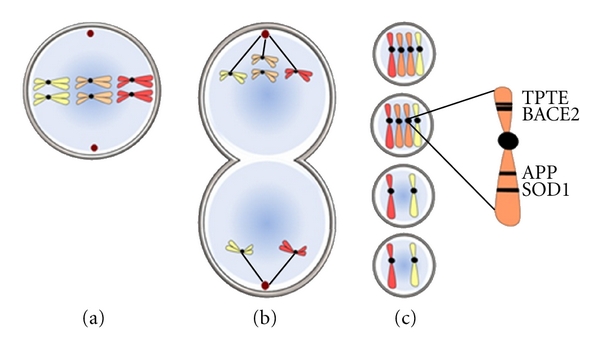
Chromosomal nondisjunction. (a) Most often Down syndrome (DS) occurs as an error in meiosis I (usually in the oocyte). Chromosomal nondisjunction, or improper segregation of chromosome 21 (the smallest autosome; orange), results in one precursor cell having 2 copies (b), upper half) while the other has zero (b), lower half). (c) Meiosis II then proceeds, with the outcome being two gametes that possess an extra copy of chromosome 21 which, after fusion with another gamete, bears 3 copies of chromosome 21; the genetic condition known as DS or trisomy 21. Also produced in this process are two nonviable gametes that possess zero copies of chromosome 21 (bottom).

According to the CDC, 1 in 700 infants born have Down syndrome (DS), approximately 400,000 people in the US and 6 million people world-wide. DS is caused by an extra copy of chromosome 21 that arises during gametogenesis. In 95% of cases, this occurs as the result of chromosomal nondisjunction [1]. This is usually due to improper segregation of chromosomes into daughter cells during meiosis I (Figure 1), although nondisjunction in meiosis II also occurs. This results in gametes that have two copies of chromosome 21 (HSA 21), and upon fusion with another gamete, results in trisomy 21. Although HSA 21 is the smallest human autosome, the chromosome encodes more than 400 known genes [2], a number that may increase with further study. Less frequently, DS occurs due to somatic mosaicism or translocations [1]. DS presents with an easily recognizable phenotype, including a characteristic set of facial features, delayed development, and varying levels of intellectual disability, shortened stature, muscle hypotonia, joint laxity, AD-like neuropathology, and a heterogeneous range of other traits.

 Advances in health care have led to improved longevity for individuals with DS, with the expected lifespan now approaching 60 years. While advanced maternal age is the only well-documented risk factor for DS [3], many socioeconomic and environmental factors that are difficult to evaluate may affect prevalence and survivability. With aging, the DS population faces an entirely different set of challenges. By the late 1800s, it was documented that individuals with DS develop plaque and tangle neuropathology that is similar to the one described in 1906 by Alois Alzheimer and is now known as Alzheimer's disease (AD) pathology (reviewed in [4]). AD is a disease that has progressed in our social consciousness from a peculiar rarity less than half a century ago to one of the greatest public health concerns of our generation [5]. We now know that essentially all individuals with DS develop AD-like pathology by the fourth decade of life. Interestingly, this predated the finding that an extra copy of chromosome 21 causes DS by almost 50 years [6]. Clues as to how this predisposes individuals with DS to AD-like pathology became more clear with the finding that HSA 21 harbors the genes for the amyloid precursor protein (APP) and BACE2, two genes directly implicated in AD pathogenesis.

Alzheimer's disease is a devastating disease and is a growing public health concern as our population ages. The most common form of dementia among the elderly, AD is already taking a toll on our health care system, and many families struggle to provide necessary care. AD manifests as a progressive cognitive decline, including memory loss, speech dysfunction, and impaired spatial orientation, as well as a host of other symptoms [7]. In the general population, AD manifests in two forms: an autosomal dominant early onset form of the disease, familial AD (FAD), that accounts for less than 1% of disease cases, and the more common sporadic form of late-onset AD. Age of onset distinguishes the two groups, but clinical presentation and neuropathology are identical [8]. Thus, studying FAD gene mutations has provided insight into the molecular mechanisms that lead to neuropathology [9–12], even though the process may begin as much as 20 years before the patient begins to present clinically with symptoms [13].

## 2. The Molecular Neurobiology and Histopathology of AD

AD is characterized by the presence of two neuropathological lesions, extracellular plaques composed largely of a 40–42 amino acid peptide called *β*-amyloid (A*β*), and intracellular tangles and striated neuropil threads composed of a hyperphosphorylated form of the cytoskeletal protein tau [14–16]. Synapse loss in areas of the brain vital for learning and memory correlates with a patient's performance on cognitive tests even in cases of mild AD and precedes neuronal loss, which becomes prevalent in mild-AD. [17, 18]. This neuropathology eventually encompasses most of the brain, which ultimately becomes atrophied, with enlarged ventricles and significantly less overall brain weight than a comparatively aged healthy brain.

**Figure 2:**
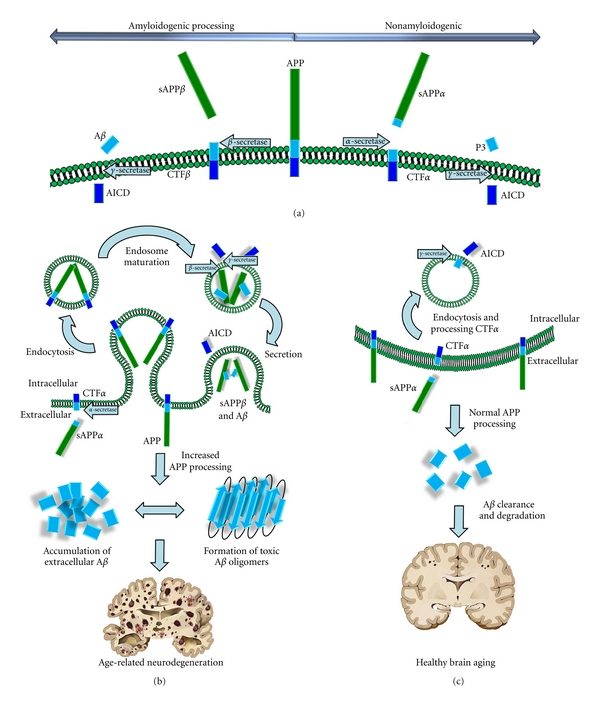
APP processing and imbalance in age-related neurodegeneration. (a) The amyloid precursor protein is processed either by an amyloidogenic pathway (left) or a canonical pathway (right). Canonical processing by *α*-secretase results in secretion of a large extracellular fragment, sAPP*α*. Importantly, this cleavage occurs within the A*β* peptide fragment (light blue), preventing its formation. A membrane bound C-terminal fragment, CTF*α*, then becomes a substrate for *γ*-secretase. This cleavage occurs within the membrane, releasing a short extracellular p3 peptide, and the APP intracellular domain (AICD, dark blue). Amyloidogenic processing occurs as APP interacts with *β*-secretase, or BACE, in the endocytic pathway. This generates the secreted sAPP*β*, and a longer C-terminal fragment, CTF*β*; *γ*-secretase cleavage of this fragment generates A*β* and AICD. (b) In Down syndrome, the overexpression of APP on the cellular surface results in increased amounts of APP being endocytosed. In mature endosomes, BACE (an enzyme that is more active at acidic pH) then cleaves APP resulting in increased amounts of CTF*β* and A*β* peptide (light blue) being secreted outside the cell. Increased extracellular accumulation of toxic A*β* species, particularly A*β*_42_, results in the formation of A*β* oligomers. These oligomers then overwhelm the brains capacity for clearance and degradation and form extracellular plaques, ultimately leading to neurodegeneration and severe brain atrophy. (c) Normally, most APP is cleaved by the *α*-secretase, secreting sAPP*α*. CTF*α* is endocytosed and then processed by *γ*-secretase, resulting in formation of the p3 peptide, which is secreted, and releasing the AICD into the cytosol. BACE processing of APP does occur to generate A*β* (blue), but these are degraded and cleared. While few small plaques may accumulate with aging, they are much smaller and fewer in number than those associated with disease.

Characterization of genomic mutations present in early onset FAD led to the amyloid cascade hypothesis [19]. The amyloid precursor protein (APP) is a ubiquitously expressed type 1 transmembrane protein similar in structure to a receptor [20], but after years of intense study no universally accepted ligands have been identified [21]. The processing of the protein is now known in considerable detail [22–24] (Figure 2(a)). Nonamyloidogenic APP processing by *α*-secretase on the cell surface results in cleavage within the A*β* peptide fragment thereby abrogating A*β* peptide formation and resulting in secretion of a large fragment, sAPP*α*. The resultant transmembrane c-terminal fragment (CTF*α*) is a substrate for *γ*-secretase processing, but results in secretion of a peptide fragment much smaller than A*β*, called p3. Cleavage of APP by a transmembrane aspartyl protease, *β*-site APP site cleaving enzyme (BACE), occurs in the endocytic pathway (Figure 2(b)) and results in the transmembrane fragment CTF*β*. Subsequent cleavage in the transmembrane domain of CTF*β* by *γ*-secretase generates secreted A*β* peptide fragments 38–43 residues in length. Cleavage of either CTF*α* or CTF*β* by *γ*-secretase also results in the generation of a small, cytosolic fragment (AICD) of poorly understood function. FAD-linked mutations in APP generally result in an increase in A*β*_42_ production [25, 26]; this is thought to be the most toxic peptide species generated by this noncanonical APP processing pathway and leads to aggregation and formation of higher order structures including oligomers (reviewed in [27]) that damage neurons and induce pathogenesis [28]. This slightly longer peptide fragment is more hydrophobic and is thought to seed neuritic plaque deposition by causing aggregation of other species that are more soluble, such as A*β*_40_ [29, 30].

The 400 known genes on HSA 21 represent many protein families and diverse functions, including the transmembrane phosphatase with tensin homology (TPTE) and superoxide dismutase (SOD1). HSA 21 harbors at least two genes implicated in the development of AD-like pathology (Figure 1(c)). The first is APP, the substrate from which the pathogenic A*β* peptide is derived. The second is BACE2, an aspartyl protease with ~65% sequence homology to BACE1, the major form of *β*-secretase in the brain. BACE1 was originally discovered by multiple groups as the primary *β*-secretase responsible for A*β* generation in the brain [21, 31–34], and the homologue BACE2 was discovered shortly thereafter [35, 36]. The *β*-secretases belong to the pepsin family of aspartyl proteases and are the only transmembrane domain containing members. The BACE1 gene is found on chromosome 11 and encodes a 501 amino acid protein, while the BACE2 protein is found on chromosome 21 and encodes a 518 amino acid protein (reviewed in [37]). Like other aspartyl proteases, both BACE1 and BACE2 have an N-terminal prodomain that is cleaved by a furin-like protease or through autoproteolytic cleavage [38] to generate the mature enzyme. One of the primary differences between the enzymes occurs within the C-terminal portion of the proteins, with the BACE1 active-site containing 3 disulfide bonds, while BACE2 has 2 [39]. 

## 3. ***β***-Secretases and Neuropathology

Since its discovery little more than a decade ago, a vast body of work has amassed supporting the role of BACE1 in AD. BACE1 activity has been established as the rate-limiting step in formation of the A*β*-peptide. BACE1 levels increase slightly during the normal aging process [40, 41], but it is well established that both BACE1 protein and enzymatic activity are further increased in the AD brain [42–44]. In the Swedish familial form of AD, an APP mutation at the *β*-site makes the protein a more efficient substrate for BACE, resulting in early onset dementia and a more rapid disease progression [45]. Importantly, BACE1 knockout prevents formation of the A*β* peptide in vivo, a finding that solidly supports BACE1 as the major *β*-secretase in the brain, and a prime therapeutic target for AD [46]. Although phenotypic changes in BACE1 knockout mice are subtle, it is likely that BACE1 is involved in myelination [47, 48] and is important during development and following traumatic brain injury [48, 49]. Unlike in AD, BACE1 activity in DS does not appear to be significantly increased [50]. While some reports indicate a trend toward an increase, the absence of a robust effect likely indicates that the overexpressed APP is more important for driving AD-like pathology in DS than an increase in enzymatic activity, [50–52] although other cellular processes may be involved [53].

Because BACE2 is located on chromosome 21 and initial reports indicated an ability to generate the A*β* peptide from APP [54], it seemed plausible that this enzymatic activity might contribute to AD pathology in DS [35]. Recent evidence indicates that BACE1 and BACE2 activities and expression are highly correlated in the brain, including in individuals with DS [50]. However, significant effort from multiple groups has uncovered little evidence to support a role for BACE2 in driving the disease process. While BACE2 mRNA is increased in DS [55], posttranscriptional regulatory mechanisms either prevent an increase in translation or affect flux of the protein by increasing the rate of degradation. Many groups have reported that levels of BACE2 protein in the DS brain are comparable to control brains in various brain regions [50, 55–57]. Even though structural studies indicate that the active sites of both BACE1 and BACE2 are very similar [39], overexpression studies of BACE2 in both primary and immortalized cell culture models generally result in decreased A*β* production [58]. Other studies indicate that BACE2 has a higher propensity to cleave APP downstream from the BACE1 protease site, actually abrogating A*β* formation [37, 58, 59]. In vivo studies using transgenic mice that overexpress BACE2 alone [60] or cooverexpress both BACE2 and APP [61] do not show a resultant increase in A*β* peptide in the brain. These findings taken together indicate that BACE2 is probably not responsible for AD pathology in the DS brain and, indeed, may have a protective function.

## 4. APP and A***β***

There is much debate about which characteristics confer toxicity to the A*β* peptide. The N-terminal end of the peptide, formed by *β*-secretase cleavage, is fairly heterogeneous and subject to various modifications. The C-terminus, produced by intramembrane processing of the CTF by the *γ*-secretase, yields a peptide 39–43 amino acids long, with A*β*_40_ and A*β*_42_ being the most abundant species. The peptide likely exists as a dynamic pool of forms ranging from soluble dimers through higher order oligomers that become increasingly insoluble with size and result in plaque deposition. While many of the events regarding this process are poorly understood, it is likely driven biochemically by sequestration of hydrophobic regions from the aqueous environment [62]. It is widely accepted that the 42 amino acid peptide is more hydrophobic and aggregate prone and is proposed to seed plaque formation in the brain. A*β*_42_ is the first peptide species to form extracellular deposits in the DS brain, and these deposits are abundant in brains from young individuals with DS by 12 years of age, approximately 20 years before significant A*β*_40_ and tau histopathology can be found [63].

The A*β* peptide is a fragment of APP, a transmembrane protein of unknown function. Recently, it was proposed that APP stimulates neuroprogenitor cells to develop into various glial cell lineages and could be a possible contributor to the decreased neurogenesis and delayed development seen in DS [64]. A role in the vasodilation process has also been suggested and represents a potential mechanism for APP-mediated cerebral amyloid angiopathy, a process that could contribute to early neuropathology in AD [65]. The APP gene is found in the DS obligate region, and the protein is overexpressed in the adult DS brain [50, 56]. Overexpression of APP leads to dysfunction of the endocytic system, resulting in increased turnover from the cellular surface, thereby increasing the likelihood that APP will encounter *β*-secretase and be processed via the amyloidogenic pathway [66]. This will result in more intracellular APP carboxyl-terminal fragment(s) cleaved at *β*-site(s) (CTF*β*), and in turn more A*β* will be generated in the DS brain. Given that *β*-secretase itself does not appear to specifically increase in DS [50], it would thus appear that APP overexpression is the main driver of AD-like pathology in the brains of elderly DS individuals.

## 5. Conclusion

While there are similar neuropathological changes in people with DS compared to AD, the brains of these populations are quite different. The DS brain is slower to develop and smaller at maturity than the brain of a diploid individual, weighing less than 1250 and often under 1000 grams, several hundred grams less than normal. Anatomically, the DS brain is more rounded with a distinct fore-shortened shape, and smaller frontal lobes, hippocampi, and cerebellum (reviewed in [4]). The brain in older individuals with DS is susceptible to cell loss in both cortical and subcortical regions, resulting in dysfunctions in both neurotransmitter systems and neuronal circuitry.

Emerging evidence from both fetal and adult DS tissues and animal models of DS indicates that changes at the molecular level are more wide spread than previously acknowledged. While there are about 400 known genes on chromosome 21, a meta-analysis of the transcriptome and proteome reveals that many more are affected. Several—but not all—genes on chromosome 21 were overexpressed, while expression of others was unchanged or even decreased [67]. This indicates that the in vivo state is the result of a more complex interplay of factors than a simple gene dosage effect. There may be over 300 genes that are significantly changed in DS, the majority of which are not located on chromosome 21, and many of which have known roles in early developmental processes. The role of these various changes in development and the penetrance of many of the typical phenotypes of DS is largely unknown. Recently exon tiling arrays have been used to interrogate the role of various genomic loci in DS features, using rare segmental trisomies [68]. Importantly, this work highlights that the obligate region of chromosome 21 is more heterogenous than anticipated and may not exist at all, as individuals with segmental trisomies can still present with a moderate to severe DS phenotype. One of the patients characterized, a 65-year old without an additional copy of APP, did not have dementia or indication of amyloid accumulation when assessed by brain imaging, supporting a causative role for APP overexpression in neuropathology in DS [68].

In the general population, a definitive neuropathological diagnosis of AD requires that the classical hallmarks of AD, namely, neuritic plaques and neurofibrillary tangles, to be present along with a clinical history of dementia. Although this characteristic AD-like pathology is present by the fourth decade of life, not all individuals with DS develop dementia, even with complete trisomy 21 [69]. Even though changes in cognitive ability and social withdrawal are often reported by caregivers of middle-aged persons with DS, there is some controversy about whether this represents a clinically defined dementia [4]. Prevalence rates for dementia in DS vary considerably between studies, but are approximately 15%, slightly higher than that in the general population; however, in DS, the dementia occurs at significantly younger ages (reviewed in [70]). Cognitive testing for DS has proven difficult, which is not surprising given the wide range of intellectual disabilities presented. Also, because there is often little cognitive data for individual patients before their decline, establishing a cognitive baseline is not often possible for individuals. These issues at the individual level make it difficult to elucidate effects in groups, resulting in floor effects plaguing cognitive tests, and difficulty making conclusions regarding population-wide affects in DS [71, 72]. A better understanding of the cognitive strengths and weaknesses of individuals with DS (reviewed in [73]) and how these change over time represents a huge need for the DS community. Recently, much effort has been put into developing cognitive tests specifically for DS, such as the Arizona Cognitive Test Battery [74]. These testing methods that can be used across a wide range of ages and cultures with little dependence on language skill are an important step forward. In addition, both functional and cognitive abilities are assessed, which are particularly useful for longitudinal studies of basic cognitive ability in persons with DS and discerning if they do indeed develop AD. As a diagnosis of AD requires both neuropathology and dementia, it is important for many reasons that we know the clinical consequences of AD-like pathology in DS versus the non-DS population.

DS is commonly recognized as a model for AD pathology, and is very much proof of principle for the amyloid cascade hypothesis, because the additional copy of APP in DS results in pathology long before it occurs in the general population. As such, if the progression to dementia is delayed or absent in DS, this may help us elucidate a therapeutic strategy that may be applicable to patients with familial or sporadic AD as well. Therapies to treat Alzheimer's disease in both the DS population and general population are limited. No pharmacological agents have been described that are able to alter disease progression. Symptoms may be improved by a cholinesterase inhibitor (donepezil, rivastigmine, galantamine), or NMDA receptor antagonist (memantine) (reviewed in [75]). Current goals include determining which biomarkers are indicative of the disease process years before development of pathology, which may lead to therapeutics designed to alter the disease process. Still, many questions remain. Although the pathway driving the degenerative process in DS may be different than the one in familial or sporadic AD, and is likely fueled by substrate (APP) overexpression, the neuropathological hallmarks of the disease are the same. How much do these pathways overlap compared to sporadic AD that occurs in the general population? Are there factors responsible for controlling progress for dementia that are altered in DS, and are these a direct or indirect consequence of an extra copy of HSA 21? Many non-DS individuals who have been followed longitudinally and come to autopsy have sufficient neuritic plaques and neurofibrillary tangles to meet the critera for a neuropathological diagnosis of AD, yet there is no evidence to suggest they experienced cognitive impairment or decline, and so are referred to as preclinical AD [76]. Although it is possible that they would eventually progress to dementia, it is also possible that these individuals exhibit a compensatory mechanism that allows them to endure this neuropathology relatively unscathed. A similar mechanism may be at work in DS.

While there is much to learn, developing and executing longitudinal studies for persons with DS is difficult, and success will depend on an integrated, informed, and motivated network of parents and caregivers of persons with DS, medical professionals that better understand the range of primary and secondary complications that result from DS, and involvement and outreach from the research community. This process has already begun as two goals stemming from the National Institutes of Health's Research Plan on Down syndrome will be realized within the next year. The first is the development and testing of a national registry for DS, and the second is the establishment of a consortium to bring clinicians and researchers together [77]. These are exciting steps for the DS community and hopefully just the beginning of many resources that will benefit individuals with DS. However, there are still many challenges and areas where improvements are needed, including identifying socioeconomic factors that impact the early development and increased risk of mortality among certain ethnicities; developing learning tools and programs specifically for intellectual disabilities; educating families and healthcare personnel so individualized health plans and testing for routine and secondary afflictions can be monitored routinely; performing routine functional and cognitive testing prior to decline; and finally, using therapeutics for age-related concerns such as depression and AD.
